# Revisiting the Therapeutic Potential of *Bothrops jararaca* Venom: Screening for Novel Activities Using Connectivity Mapping

**DOI:** 10.3390/toxins10020069

**Published:** 2018-02-06

**Authors:** Carolina Alves Nicolau, Alyson Prorock, Yongde Bao, Ana Gisele da Costa Neves-Ferreira, Richard Hemmi Valente, Jay William Fox

**Affiliations:** 1 Laboratory of Toxinology, Oswaldo Cruz Institute, FIOCRUZ, Rio de Janeiro, RJ 21040-900, Brazil; carolnicolau.bio@gmail.com (C.A.N.); anag@ioc.fiocruz.br (A.G.d.C.N.-F.); 2 National Institute of Science and Technology on Toxins (INCTTOX), CNPq, Brasília, DF 71605-170, Brazil; 3 Department of Microbiology, Immunology and Cancer Biology, University of Virginia, Charlottesville, VA 22908, USA; ajp7x@virginia.edu (A.P.); yb8d@virginia.edu (Y.B.)

**Keywords:** *Bothrops jararaca*, therapeutic potential, connectivity map, drug discovery, biosimilar drugs

## Abstract

Snake venoms are sources of molecules with proven and potential therapeutic applications. However, most activities assayed in venoms (or their components) are of hemorrhagic, hypotensive, edematogenic, neurotoxic or myotoxic natures. Thus, other relevant activities might remain unknown. Using functional genomics coupled to the connectivity map (C-map) approach, we undertook a wide range indirect search for biological activities within the venom of the South American pit viper *Bothrops jararaca*. For that effect, venom was incubated with human breast adenocarcinoma cell line (MCF7) followed by RNA extraction and gene expression analysis. A list of 90 differentially expressed genes was submitted to biosimilar drug discovery based on pattern recognition. Among the 100 highest-ranked positively correlated drugs, only the antihypertensive, antimicrobial (both antibiotic and antiparasitic), and antitumor classes had been previously reported for *B. jararaca* venom. The majority of drug classes identified were related to (1) antimicrobial activity; (2) treatment of neuropsychiatric illnesses (Parkinson’s disease, schizophrenia, depression, and epilepsy); (3) treatment of cardiovascular diseases, and (4) anti-inflammatory action. The C-map results also indicated that *B. jararaca* venom may have components that target G-protein-coupled receptors (muscarinic, serotonergic, histaminergic, dopaminergic, GABA, and adrenergic) and ion channels. Although validation experiments are still necessary, the C-map correlation to drugs with activities previously linked to snake venoms supports the efficacy of this strategy as a broad-spectrum approach for biological activity screening, and rekindles the snake venom-based search for new therapeutic agents.

## 1. Introduction

The development of therapeutic drugs such as the antihypertensive Captopril^®^ [1,2] and the anticoagulant Exanta^®^ (also known as ximelagatran) [3] can be traced back to the study of isolated snake venom components and their biological roles during envenomation. Over the years, venoms, and fractions thereof, have displayed several biological activities/applications, including antibacterial [4,5,6,7,8,9,10], antiprotozoarian [7,11,12,13,14,15,16], antimeasles [17], antiviral (human immunodeficiency virus) [18,19], analgesic [20,21,22,23,24], and for the treatment of multiple sclerosis [25]. It is important to note that some of those aforementioned activities can be related not only to medium to high abundance specific venom toxins but also to low abundance components and, eventually, to their synergistic effects. Also, secondary effects generated by venom components should be considered; such is the case for the activation of inflammation and apoptosis pathways through the action of DAMPs (damage-associated molecular patterns), released after tissue injuries generated by the snake venom/snake venom fraction being assayed [26]. For instance, DAMPs released in the wound exudate after viperid envenomation contribute to vascular permeability mediated by TLR4 (toll-like receptor 4) [27].

The use of functional genomics (microarray techniques) to analyze the subtoxic effects, through gene expression analysis, on cell cultures treated with snake venoms and/or their components has been successfully demonstrated [28,29]. However, it is still challenging to associate signaling pathways identified through functional genomics to the pathophysiology of snakebite (assessed through well-established biochemical and biological assays, screening for hemorrhagic, hypotensive, edematogenic, neurotoxic, and myotoxic activities) [30]. Although these assays are useful in reproducing some of the effects of snakebite envenoming, activities other than those traditionally associated with snake venoms could remain unknown. Hence, without a priori knowledge, it is no simple task to identify potentially novel therapeutic activities derived from snake venoms and/or their components.

An alternative “blind” biological activity screening approach is to use the C-map (connectivity map) platform (https://portals.broadinstitute.org/cmap/). C-map consists in a public database of gene expression patterns generated from the treatment of known cell lineages with 1309 small molecules and drugs, whose pharmacological properties are well characterized [31,32]. Thus, the biological activity of the sample tested can be indirectly inferred by matching the experimental list of differentially expressed genes to the gene expression patterns present in the C-map database. A proof-of-concept for the application of C-map approach in Toxinololgy was demonstrated by treating MCF7 (Michigan cancer foundation 7) cells with *Heloderma suspectum* (Gila monster) venom or the anti-diabetic drug Byetta (developed from a peptide isolated from that same venom). As predicted, C-map analysis of differentially expressed genes in either condition displayed high positive correlation with different anti-diabetes drugs [33].

Thus, to test the feasibility of C-map analysis for biological activity screening in snake venoms, we chose the venom of the South American pit viper *Bothrops jararaca*, one of the best characterized venoms by proteomic approaches [34]. Although this venom is highly diverse, few protein classes account for around 94% of its composition [34] (Table 1). Consequently, the less abundant proteins such as hyaluronidases, cysteine-rich secretory proteins, growth factors, nucleotidases, among others, are underexplored [35,36], resulting in a lack of knowledge about their individual contributions to the snake envenoming pathology. Boldrini-França and colleagues [37] recently emphasized the importance of studying and characterizing minor components from snake venoms, since these can display different potential therapeutical applications, such as: antiparasitic, antitumor, neuroprotection, and ischemic tissue protection.

In this work, we have analyzed the gene expression of MCF7 cells treated with *B. jararaca* venom and used connectivity mapping to infer novel (therapeutic) activities potentially present in this biological sample. The majority of biosimilar drugs inferred were related to antimicrobial and anti-inflammatory activities, as well as to the treatment of neuropsychiatric and cardiovascular diseases. In short, our data rekindle the snake venom-based search for new therapeutic agents.

## 2. Results and Discussion

### 2.1. Gene Expression Analysis

MCF7 cells were used in this work since most of the C-map database information relies on assays using this cell type, due to its extensive molecular characterization and ubiquitous use as a reference cell line [32]. However, since MCF7 cells are not natural targets for snake venom components, it was not the focus of this study to make detailed associations between differentially expressed genes and snakebite envenoming. More importantly, our goal was to submit the list of up- and down-regulated genes to C-map analysis, in order to screen for a panel of biosimilar drug activities related to *B. jararaca* venom. Nonetheless, we will highlight some of the differentially expressed genes and their possible correlations with snake venom toxins.

*B. jararaca* venom induced (*p*-value < 0.01) the differential expression of 90 genes (74 up- and 16 down-regulated) in MCF7 cells. We only considered up- or down-regulated genes those displaying a log_2_ of fold-change equal or greater than 0.58 (fold change ≥1.50) or −0.58 (fold change ≤0.67), respectively, when compared to expression in the untreated cells (control). The up- and down-regulated genes are shown as supplementary material (Tables S1 and S2, respectively) and the data used to generate these tables are supplied in Tables S5 and S6.

The cytochrome P450 family, which is represented by heme-thiolate proteins [60], displayed the highest differentially expressed gene. The CYP1A1 (cytochrome P450, family 1, subfamily A, polypeptide 1) gene had a 29.6-fold increase in expression, compared to control, when MCF7 cells were treated with venom (Table S1). Among the up-regulated genes, we also identified another member of this family, CYP1B1, with 3.4-fold increase. CYP1A1 and CYP1B1 genes are involved in the metabolism of arachidonic acid generating ROS (reactive oxygen species), which is one of the triggers to initiate the apoptosis process [61]. Even though the cytochrome P450 main function is to metabolize drugs and synthesize lipids such as cholesterol and steroids [62], its high expression in MCF7 cells treated with *B. jararaca* venom could also be influenced by three venom components activities through: (i) indirect involvement in the metabolism of arachidonic acid [63] eventually released after PLA_2_ (phospholipase A_2_) metabolizes phospholipids [64]; (ii) involvement in the metabolism of arachidonic acid released by the action of bradykinin, which would be possible due to the action of BPPs (bradykinin-potentiating peptides) present in snake venoms [65]; and (iii) use of hydrogen peroxide, released by the action of venom LAAO (l-amino acid oxidase), as an oxygen donor [60]. Those activities may contribute to activation of apoptosis- and inflammatory-related pathways through the generation of ROS. In this regard, the venom from another Viperidae, *Echis carinatus*, induced an overexpression of genes associated to ROS pathways, including the cytochrome P450 enzymes, in HUVECs (human umbilical vein endothelial cells) [66]. Additionally, *B. jararaca* and *Crotalus atrox* venoms induced a significant increase in the expression of genes related to apoptosis and inflammatory pathways in HUVECs [28]. Interestingly, these authors also showed that the proteolytic activity of jararhagin, the major hemorrhagic metalloendopeptidase from *B. jararaca* venom, is mandatory for the generation of an inflammatory and pro-apoptotic response in human fibroblasts [29].

The presence of oxidative stress in MCF7 cells treated with *B. jararaca* venom is also supported by the significantly higher expression of HMOX1 (heme oxigenase 1) (Table S1), which is an enzyme involved in antioxidant response [67]. HMOX1 degrades heme releasing antioxidant agents such as carbon monoxide and biliverdin (which is further converted to the antioxidant bilirubin) [68,69]. Thus, the higher expression of HMOX1 may represent a response to the oxidative stress induced by *B. jararaca* venom.

Finally, Sunitha and co-workers [26] summarized experimental evidence from the literature for oxidative stress and inflammation induced by viper bites, as well as the apparent involvement of DAMPs, generated after SVMP (snake venom metalloendopeptidase) and PLA_2_ activities, in these processes. Recently, it has been confirmed that at least part of the inflammatory process generated after viper bites is dependent on the activation of TLR4 pathway by DAMPs [27].

Overall, it is possible that *B. jararaca* venom induces apoptosis and inflammation through different pathways. The apoptotic feature of snake venoms is likely related to secondary molecules such as H_2_O_2_ released after LAAO activity and NO (nitric oxide) production. Snake venoms such as *B. jararaca* and *B. asper* are able to induce the release of inflammatory mediators like NO [70,71,72]. Although MCF7 cells do not possess the major molecular targets of snake venoms, and do not produce cytokines, it has been demonstrated that breast cancer cells, including MCF7, express inducible NO synthase [73,74,75].

### 2.2. Connectivity Map Analysis

We submitted the MCF7/*B. jararaca* venom genomic signature (list of up- and down-regulated genes following MCF7 cells treatment with venom) to the C-map algorithm for comparison with the gene-expression profiles (signatures) generated by the treatment of different cell lineages with drugs or small molecules, also called perturbagens. In short, the algorithm returns a list of perturbagens (compounds) with score values ranging from +1.000 to −1.000, encompassing the most positively- (agonistic effect) to the most negatively-(antagonistic effect) correlated perturbagens. The C-map score is calculated by a combination of the up and down scores (which represent the absolute enrichment of the list of up- and down-regulated genes, respectively) submitted to the algorithm when compared to the signatures induced by the perturbagen. The C-map score reflects how well the genomic signature induced by the assayed sample correlates with the perturbagens’ genomic signatures deposited in the database. In the original publication [31], no statistical treatment has been envisaged following the C-map score calculation. Therefore, to ensure low false discovery rates, a reasonable alternative could be to consider only the highest C-map score values (e.g., >+0.900 or <−0.900). However, when looking at the data from the literature where, following C-map analysis, biological validation assays have been performed, C-map score values for confirmed hits were as low as 0.530 [31] and −0.777 [76]. The present work established an arbitrary C-map score threshold of 0.600. On one hand, we acknowledge that, at some instances, this could eventually generate a more speculative discussion. On the other hand, our C-map results (Table S3) displayed positive hits to most of the published biological activities (related to possible therapeutical applications) directly associated to different snake venoms (Table 2), indicating that, as expected, the biological significance of the results has not been impaired by a less stringent cut-off value.

Considering only genomic signatures generated by MCF7 cells treated with known drugs, we identified 792 positive correlations, sometimes also described as “agonist-related” activities (Table S7). The top-100 positively correlated drugs are shown in Table S3, and some of them will be discussed below. Additionally, we have rearranged the data from Table S3 according to the major findings and their applications: antimicrobial, anti-inflammatory, and treatment of neuropsychiatric or cardiovascular disorders (Table 3, Table 4, Table 5 and Table 6). The top-20 negatively correlated signatures (“antagonist-related”) are shown in Table S4.

#### 2.2.1. Major Drug Classes Positively Correlated to Venom through C-Map Analysis

##### Antimicrobial Activity

Our biosimilar drug discovery study revealed 20 antimicrobial molecules (Table 3), of which 16 were antibiotics and 4 were antiparasitics (antimalarial, antifungal/antiprotozoal, and antischistosomal).

Antibiotic activity has already been reported for *B. jararaca* venom against Gram-negative and Gram-positive bacteria [4], as well as in other venoms from the *Bothrops* genus [8,9,10]. Additionally, all these studies have associated the antibiotic activity of snake venoms to LAAO or PLA_2_, even though their mechanism of action remains unclear. Both enzymes, isolated from different snake venoms (including *B. jararaca*’s) are also frequently associated with anti-parasitic action, such as trypanocidal and leishmanicidal [4,11,13,14,77,79,80].

The second highest positively-correlated drug identified through C-map was primaquine, the only antimalarial drug available to treat malaria relapse caused by *Plasmodium vivax* [102,103]. This parasite presents a dormant stage (hypnozoite), which remains in the liver, creating a persistent reservoir of infection by subsequently reactivating blood-stage infections [104]. Although primaquine is the current treatment against hypnozoite forms of *P. vivax*, the drug has limited therapeutic efficacy [105] and is toxic to glucose-6-phosphate dehydrogenase deficient patients, due to the risk of hemolytic anemia [106]. Also, studies have indicated that some hypnozoites may be resistant to primaquine [107]. Thus, the development of more effective antimalarial treatments against hypnozoite stages of *P. vivax* is highly desirable [105]. Furthermore, halofantrine, another antimalarial which acts similarly to chloroquine by forming toxic complexes with ferritoporphyrin IX, thereby damaging the membrane of the parasite [108,109,110], was also inferred by our C-map data (Table 3).

Although anti-parasitic (*Leishmania amazonensis*, *L. chagas*, *L. infantum*, *L. major*, *Trypanosoma cruzi*, and *Plasmodium falciparum*) activities have already been reported for venoms (and fractions thereof) from different *Bothrops* genus snakes [14,15,16,78,79,81,82,83], an anti-*Plasmodium* activity had not yet been described specifically for *B. jararaca* venom. However, isolated PLA_2_ from snake venoms belonging to different genera, including the genus *Bothrops*, displayed anti-*Plasmodium* activity [12,16,84,85]. It is noteworthy that primaquine induces the expression of CYP1A1 [111], which was the most up-regulated gene identified in this study.

The potential antimalarial activity herein identified may also reflect an effect of HMOX-1, coded by the third most up-regulated gene identified in this work, through heme catabolism (Table S1). HMOX-1 is able to prevent apoptosis through TNF (tumor necrosis factor) pathway in *Plasmodium*-infected hepatocytes [67]. Studies have indicated that heme might have an important role in *Plasmodium* survival, especially in the mosquito and in the liver stages of infection, since the parasite is able to synthesize heme, in addition to its capability to obtain heme from the infected erythrocyte [112,113]. Furthermore, carbon monoxide released as a consequence of HMOX-1 enzymatic activity precludes the start of cerebral malaria through binding to hemoglobin released from the cells, thus preventing heme release [114,115].

In summary, some findings of this work corroborate the presence of antimalarial component(s) in *B. jararaca* venom. They consist of (i) the up-regulation of HMOX1 gene (Table S1) and (ii) the C-map analysis that led to the biosimilar drug discovery of the antimalarials primaquine and halofantrine (Table S3).

##### Neuropsychiatric Illnesses

C-map analysis associated *B. jararaca* venom to 19 drugs used in the treatment of neuropsychiatric disorders; among those, ten antipsychotics, three antidepressants, four anticonvulsants, and two antiparkinsonian drugs (Table 4). These compounds, especially the antipsychotics, usually act on muscarinic, adrenergic, dopaminergic, serotonergic, and/or histaminergic postsynaptic receptors [116,117,118,119,120]. The aforementioned receptors belong to the GPCR (G-protein-coupled receptor) family [121] and they are involved in different cell signal transduction pathways induced by hormones and neurotransmitters [122]. Additionally, the metabotropic glutamate and GABA_B_ (gamma-aminobutyric acid, class B) receptors are also described as potential targets for treatment of multiple disorders related to the CNS (central nervous system), such as depression, anxiety disorders, schizophrenia, epilepsy, Alzheimer’s, and Parkinson’s diseases [123,124,125,126].

The potential of snake venom components to treat CNS disorders [127] may be partially explained by the presence of neurotoxins that target muscarinic receptors [128,129,130,131] and/or other families of G-protein-coupled receptors [132,133,134,135]. Three finger toxins are widely described in venoms of members of the Elapidae family; they act on a great variety of targets, including: (i) muscle nicotinic acetylcholine receptor; (ii) neuronal nicotinic receptor; (iii) muscarinic receptor (agonist or antagonist); (iv) acetylcholinesterase (inhibitor); (v) calcium channel; (vi) potassium channel-interacting protein; and (vii) β1- and β2-adrenergic receptors [58]. Although 3FTX are primarily described for Elapidae venoms, they were recently identified, albeit in low abundance, in the venom of *B. jararaca* (Viperidae family) [34]. Thus, it is possible that 3FTX are responsible, at least partially, for the potential of a *B. jararaca* venom isolated component to treat CNS disorders. CRISPs (cysteine-rich secretory proteins) present in Viperidae venoms, including *B. jararaca,* may also contribute to that effect once they target different types of ion channels as well as nicotinic acetylcholine receptors [45,136].

**Figure 1:**
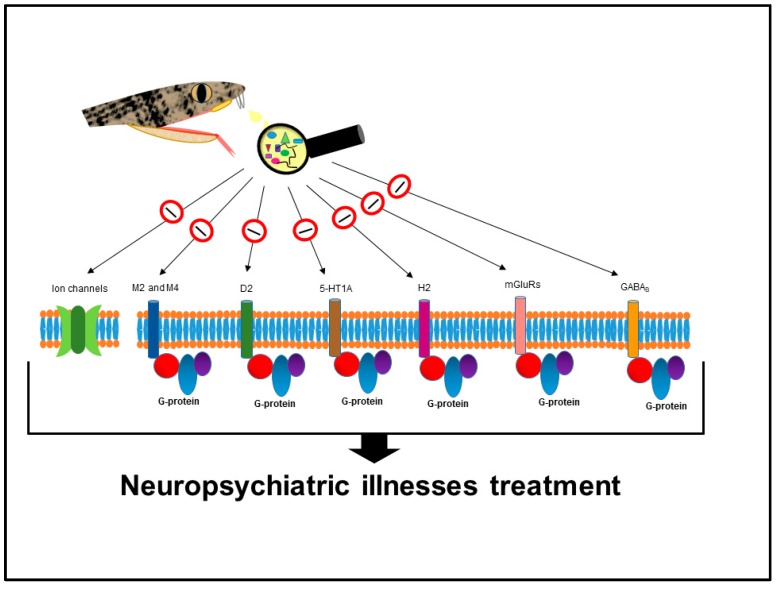
G protein–coupled receptors and ion channels potentially targeted by *B. jararaca* venom components, as hypothesized by C-map analysis. M2 and M4—subtypes 2 and 4 of muscarinic receptors; D2—subtype 2 of dopaminergic receptor; 5-HT1A—subtype 1A of 5-hydroxytryptamine serotonergic receptor; H2—subtype 2 of histaminic receptor; mGluRs—metabotropic glutamate receptors; GABA_B_—subtype B of gamma-aminobutyric acid receptor.

Different drug classes to treat neuropsychiatric illnesses have been associated to the venom; their respective targets are illustrated in Figure 1.

Antipsychotics: Antipsychotics are commonly used to treat schizophrenia primarily through dopamine receptors (especially D2) inhibition [137]. However, they also display varied affinities for serotonin, cholinergic, adrenergic, and histamine receptors [138,139]. The antipsychotics are classified in two categories, typical and atypical. Members of the former category induce high EPS (extrapyramidal side effects) such as acute dystonia, akathisia, parkinsonism, and tardive dyskinesia [140] whereas the atypical ones cause fewer EPS [141]. Clozapine was the only atypical antipsychotic drug identified in the present work. This drug is characterized by a low affinity to dopamine receptors but high affinity for 5HT_2_ (5-hydroxytryptamine, type 2) serotonin receptor [141,142]. Although clozapine is not the first drug of choice against schizophrenia, it is frequently used to treat drug resistance cases, when the typical antipsychotics have not worked [143,144].

Anticonvulsants: Anticonvulsants are used to treat epilepsy and seizures. Epilepsy is a multifactor neurological disorder characterized by a dysfunction in the speed and intensity of the electrical neuronal discharges leading to unprovoked seizures. Antiepileptic drugs can act in distinct manners: (i) by blocking ion channels, such as voltage activated sodium and T-type calcium channels, and/or excitatory amino acids receptors; (ii) by improving the GABA activity as a brain inhibitor [145]. We identified anticonvulsant drugs that target all those pathways: calcium channels (trimethadione), sodium channels (carbamazepine), and GABA (valproic acid). Additionally, we identified a carbonic anhydrase inhibitor (diclofenamide), which is primarily used to treat glaucoma [146]; however, it might be also used to treat epilepsy since the inhibition of carbonic anhydrase, and the consequent increase in brain CO_2_ level, is a known indirect pathway for epilepsy treatment [147].

Antidepressants: Symptoms of depression are common in medically sick people. However, only a few patients actually undergo a major depressive disorder. This disorder is characterized by disturbances in mood, appetite, and sleep as well as psychomotor compromise, fatigue, and suicidal thoughts, among others [148]. Dysfunctions of norepinephrine and serotonin neurotransmission are frequent in depression and anxiety disorders, which may be explained by the involvement of this neurotransmitter systems in the modulation of other neurobiological systems compromised by this illness [149]. Thus, the antidepressant drugs usually have potent effects on central noradrenergic and serotonergic systems and, in the case of the monoamine oxidase inhibitors, dopaminergic systems as well [150]. Regarding the antidepressants identified in this work, they act by inhibiting α_2_-adrenoceptor receptor (mianserin), serotonin (5-HT) reuptake (paroxetine), and monoamine oxidase A (pirlindole).

Parkinson’s Disease Treatment: Parkinson’s is a neurodegenerative disorder characterized by a progressive death of dopamine neurons leading to motor disturbances such as muscular rigidity, bradykinesia, and tremor [151,152]. The majority of antiparkinsonian drugs target serotonergic (5-HT1A) and dopaminergic (D2) receptors [153]; such is the case for lisuride, herein identified (Table 4). On the other hand, metixene, also identified in this work, is an anticholinergic drug [154]. As mentioned above (Section 2.2.1—Neuropsychiatric Illnesses) some neurotoxins have affinity for the muscarinic receptors [58]; this might contribute for the potential presence of antiparkinsonian activity in *B. jararaca* venom. Additionally, it has been shown that a tripeptide (Glu-Val-Trp) isolated from the venom of *Bothrops atrox* has the potential to decrease apoptosis in a classic model of Parkinson’s disease [92]. Considering that the compositions of *B. atrox* and *B. jararaca* venoms are related [155], the presence of this peptide and its neuroprotective activity in the venom of *B. jararaca* should be further investigated.

On the other hand, Parkinson’s disease patients typically display an accumulation of phosphorylated extracellular protein aggregates. Thus, some authors have suggested that a snake venom metalloendopeptidase, displaying a basic isoelectric point, should be able to cleave these highly phosphorylated protein aggregates, helping to slow the progression of the disease [156].

##### Cardiovascular Related Disorders

C-map analysis ascribed, with high positive correlation scores, antihypertensive and vasodilator activities amongst seven different drugs (Table 5). Those activities are usually associated to BPPs [1], which act by blocking the ACE (angiotensin-converting enzyme) [157,158], and had their structure used as a scaffold for development of the anti-hypertensive drug Captopril [2]. Although the hypotensive activity of BPPs is generally associated to ACE inhibition [157,158], BPP 5a from *B. jararaca* venom induced hypotension through muscarinic and bradykinin receptors [86], both present in MCF7 cells [159,160]. Thus, at least part of the antihypertensive activity, indirectly identified through C-map, might be related to BPP 5a. On the other hand, the antihypertensive drugs identified through C-map belong to the alpha-adrenergic blocker (phenoxybenzamine), thiazide diuretic (hydroflumethiazide), and thiazide-like diuretic (clopamide and metolazone) classes [161].

We also identified beta-1 and/or beta-2 blockers drugs (practolol and sotalol, Table 5), that are usually used to treat arrhythmias. These results suggest that *B. jararaca* venom could be a source of molecules acting on beta adrenergic receptors, similarly to beta-cardiotoxin, from *Ophiophagus hannah* venom, which blocks both beta-1 and beta-2 receptors [162]. Interestingly, we also identified beta-1 and beta-2 agonist drugs to treat heart failure/cardiogenic shock and bradycardia, respectively (Table 5).

It is important to stress that there are other snake venoms compounds such as natriuretic peptides, L-type calcium channels blockers, sarafatoxins, and vascular endothelial growth factors that display cardiovascular effects (reviewed in [163,164,165,166]). Two recent works have demonstrated the vasorelaxant effect (which is likely due to the inducing of NO production) of *Montivipera bornmuelleri* [167] and *Crotalus durissus cascavella* [168] venoms, indicating their therapeutic potential in the treatment of cardiovascular diseases such as hypertension.

**Figure 2:**
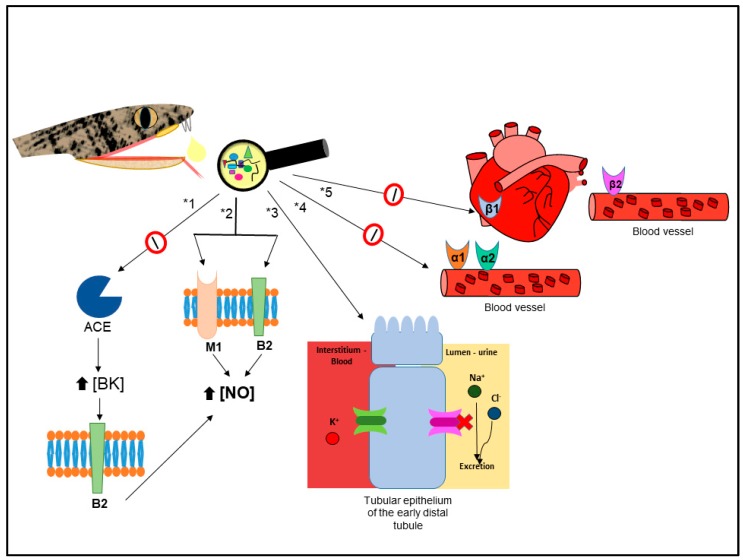
Schematic representation of established and hypothesized (this work) mechanisms of action that contribute to the overall antihypertensive effect of *B. jararaca* venom. ACE—Angiotensin-converting enzyme; BK—bradykinin; B2—subtype 2 of bradykinin receptor; M1–subtype 1 of muscarinic receptor; NO–nitric oxide; α1—subtype 1 of the α-adrenergic receptor; α2–subtype 2 of the α-adrenergic receptor; β1—subtype 1 of the β-adrenergic receptor; β2—subtype 2 of the β-adrenergic receptor; *1—antihypertensive pathway based on [2]; *2—antihypertensive pathway based on [118]; *3 to *5—hypotheses, raised after C-map analysis, suggesting that *B. jararaca* venom may present: (*3) components acting similarly to thiazide/thiazide-like molecules; (*4) α-adrenergic receptor blockers; and (*5) components inhibiting both β1 and β2-adrenergic receptors contributing to the antihypertensive effect.

All considered, it is possible that the known antihypertensive activity of *B. jararaca* venom, as well as its potential to treat other cardiovascular related disorders, is more complex than the actual perception, being related to different molecules and/or mechanisms of action, as briefly proposed in Figure 2.

##### Anti-Inflammatory

The anti-inflammatory drug Sulindac displayed the third highest positive correlation with *B. jararaca* venom effects (Table 6). Although this activity was indirectly identified 11 times among the top-100 drugs, its presence in snake venoms is unexpected, since snake venom toxins usually have pro-inflammatory effects [26,29,169,170,171,172,173]. However, this activity was recently reported for a cytotoxic protein present in the venom of *Naja naja* [93], as well as for a known analgesic peptide isolated from *Naja naja atra* venom [94]. A possible explanation would be an indirect action of *B. jararaca* venom inducing the overexpression of HMOX1, which is able to degrade proinflammatory free heme, generating carbon monoxide, iron, and biliverdin [68]. Additionally, both carbon monoxide and biliverdin, as well as its final product bilirubin, have already been described as anti-inflammatory agents [174,175,176,177,178,179].

##### Other Relevant Potential Applications

Novel Anticancer Drugs: Antitumor activity, herein associated to three drugs against different tumor cell lineages (Table S3), has previously been reported for *B. jararaca* venom [87]. The antitumor activity of snake venoms may be partially due to LAAO activity. Costa and colleagues recently published a review highlighting the antitumor potential of LAAO [88]. It is hypothesized that LAAO binds preferentially to the tumor cell surface, catalyzes the release of H_2_O_2_ which, once accumulated, induces oxidative stress leading to apoptosis [89]. Recently, Fung and co-workers [90] investigated the molecular mechanisms of antitumor effect of LAAO from *Ophiophagus hannah* through gene expression analysis of MCF7 cells. They also observed a significant increase in expression of CYP1A1 and, to a lesser extent, of CYP1B1. The authors suggested that both the direct cytotoxic effect of H_2_O_2_ released by LAAO and the oxidative stress are likely the major leading causes of apoptosis and cell death. Nevertheless, another work [91] observed that rusvinoxidase (LAAO from Russell’s viper venom) induced apoptosis in MCF7 cells through both extrinsic and intrinsic pathways, which supports the hypothesis for different pathways leading to apoptosis in tumor cells. Although LAAO is probably a key player in the antitumor effect of snake venoms, other components such as SVMPs, disintegrins, PLA_2,_ and C-type lectin/lectin-like proteins are known to have antiangiogenic properties and may also influence the overall antitumor activity [180,181,182,183].

Diabetes Treatment: Through C-map analysis, we identified three drugs to treat type II diabetes mellitus (Table S3). Tolbutamide belongs to the sulfonylureas antidiabetic drug class and acts by stimulating β cells of the pancreas to release insulin through the inhibition of a potent potassium channel on the β cells membrane [184]. Furthermore, troglitazone and rosiglitazone belong to the thiazolidinedione drug class which acts as an agonist of peroxisome proliferator-activated receptors (specifically PPARγ). This class of antidiabetic drug influences free fatty acid flux, thus reducing insulin resistance and blood glucose levels [185].

“Diabetes mellitus is a group of metabolic diseases characterized by hyperglycemia resulting from defects in insulin secretion, insulin action, or both” [186]. Insulin secretion is modulated by the action of different hormonal and neural stimuli [95,187,188], such as through the activation of G-protein-coupled receptors [189], but also through modulation of ion channel activity [190,191].

It is well known that toxins from venomous animals are able to target a great diversity of G-protein-coupled receptors, such as glucagon receptor family as well as affect membrane excitability through ionic channels modulation [95,134,192]. Thus, the identification of antidiabetic activity was not surprising since insulinotropic properties of snake venoms have already been reported for some components such as PLA_2_, serine endopeptidases, disintegrins [96], crotamine [97,193], and cardiotoxin [95]. In the case of PLA_2_, the increase in insulin secretion is likely related to cytosolic Ca^2+^ [98,99,100]. On the other hand, crotamine and cardiotoxin act on potassium and sodium ion channels, respectively [95,101,193]. It is noteworthy that Byetta^®^, a commercial antidiabetic drug, is a glucagon-like peptide-1 receptor agonist synthesized based on the peptide exendin-4, isolated from the saliva/venom of the Gila monster (*Heloderma suspecturn*) [194,195]. The potential to treat type II diabetes has also been described for components of wasp [196,197], scorpion [198,199], spider [200], and bee [201] venoms.

Gastroesophageal Reflux Disease Treatment: Gastroesophageal reflux is characterized by movement of harmful gastroduodenal contents such as gastric and bile acids into the esophagus [202]. GERD (gastroesophageal reflux disease) is a condition that causes either esophageal mucosal break, or annoying symptoms such as heartburn and regurgitation, or both [203,204]. GERD is usually treated by: (i) altering gastric contents by neutralization of acid; (ii) augmenting the antireflux barrier; (iii) improving of mucosal defense mechanisms; (iv) blocking esophageal nociceptors; or (v) modulating afferent signals and their interpretation in the brain cortex [202]. In this work, we indirectly identified one of those treatment pathways: alteration of gastric contents by neutralization of acid.

The drug lansoprazole, which showed the highest positive correlation with *B. jararaca* venom, is a proton pump inhibitor that treats GERD by blocking the gastric acid secretion [205]. However, the identification of lansoprazole may be correlated to its ability to induce the expression of CYP1A1, observed in hepatoma cell line HepG2 [206] and hepatocytes [207]. This ability has already been ascribed to primaquine [111], which was the second highest correlated drug identified (Table S3).

Moreover, H2 (histamine, type 2) receptor antagonists such as ranitidine, another drug related to the venom by C-map, can neutralize the gastric acid secretion dependent of histamine binding to H2 [208]. It has already been shown that the venom of *Bothrops moojeni* induces edema through the binding of histamine, released by the degranulation of mast cells, to H2 receptor [209]. Nevertheless, as far as we know, no compound with H2 antagonist properties has been described in snake venoms so far.

Antihistamines: We identified seven antihistamine drugs (meclozine, chlorphenamine, clemizole, carbinoxamine, ketotifen, mebhydrolin, and diphenhydramine) with good positive correlation with *B. jararaca* venom (Table S3). All these drugs display an antagonist effect on histamine receptor (H1) [210] but some of them (meclizine and mebhydrolin) have an additional anticholinergic effect. The binding of histamine to H1 receptor induces a proinflammatory response leading to many effects associated with anaphylaxis and other allergic diseases [211] such as asthma, bronchospasm, and mucosal edema [212]. The antihistaminic activity is unexpected for *B. jararaca* venom since it contains molecules (e.g., PLA_2_ and SVMPs) that are able to induce histamine release through mastocyte degranulation [213,214,215,216], leading to increased vasodilation and vascular permeability. However, considering that snake venoms can display ambivalent actions such as pro- and anti-coagulant effects or possess both agonists and antagonists of platelet aggregation [41,217], we could hypothesize that snake venoms could display antihistamine activity. It is noteworthy that MCF7 cells express both histamine H1 and H2 receptors [218].

#### 2.2.2. Major Drug Classes Negatively Correlated to Venom through C-Map Analysis

As previously mentioned, we have also generated a list of negatively correlated genomic signatures following MCF7 cells treatment with the venom (Tables S4 and S7). Although the interpretation of these results is not self-evident, we will comment on some of the hits obtained. For instance, oxymetazoline is a decongestant which acts as an alpha adrenergic agonist [219]. Since there was a negative correlation to venom, one could expect the presence of adrenergic antagonists (blockers). This is consistent with the data discussed in Section 2.2.1—Cardivascular Related Disorders, linking the venom to antihypertensive compounds. Another high-ranking hit was Trapidil, a PDGF (platelet-derived growth factor) antagonist. Although we could not find in the literature a PDGF agonist related to snake venoms, it has been shown that aggretin (a C-type lectin from *Calloselasma rhodostoma* venom [220]) phosphorylates PDGF receptor beta, leading eventually to PDGF-BB production [221]. Three anti-inflammatory- and one antihistaminic-related drugs could represent the known pro-inflammatory and histamine release activities related to bothropic venoms, which were discussed above (Section 2.2.1—Anti-Inflammatory and Section 2.2.1—Other Relevant Potential Applications: Antihistamines).

## 3. Conclusions

We aimed the exploration of novel potential therapeutic activities in *B. jararaca* venom through gene expression analysis allied to biological screening using connectivity mapping. The identification of drugs with activities (e.g., antihypertensive, antimicrobial, and antitumoral) previously reported for high abundance components of snake venoms, especially in *B. jararaca*, supported the efficacy of C-map as an unbiased exploratory approach for biological activity screening, and rekindles the snake venom-based search for new therapeutic agents. Moreover, this work indicated the existence of active venom components that could potentially be used in the treatment of other disorders (e.g., schizophrenia, depression, epilepsy, and gastroesophageal disease). However, those “newfound” activities should be assayed for in vitro and in vivo (eventually) confirmations, followed by venom fractionation in order to determine the molecular species associated to them. Furthermore, venom prefractionation could be performed and individual fractions submitted to C-map analysis; one such approach would be to assay the complex *B. jararaca* venom peptidome, recently described in the literature [34]. This peptidome is composed of hundreds of relatively short peptides (9 to 10 amino acids long, on average) that could prove a rich bioactive peptide library. In summary, the present work paves the way for further studies exploring the therapeutic potential of snake venoms by providing a rich set of novel activities to be assayed beyond the classical ones (e.g., hemorrhage, myotoxicity, hypotension).

## 4. Materials and Methods

### 4.1. Venom

Lyophilized venom, a pool from several juvenile/adult, male/female *Bothrops jararaca* specimens, was kindly provided by Instituto Butantan (São Paulo, Brazil). The access to Brazilian fauna genetic heritage was issued by the Conselho Nacional de Desenvolvimento Científico e Tecnológico (CNPq) under license number 010578/2014-5.

### 4.2. Tissue Culture

MCF7 cells were obtained from HTB022^TM^American Type Culture Collection, Manassas, VA and grown in Dulbecco’s modified Eagle medium containing 0.01 mg/mL bovine insulin and 10% fetal bovine serum. MCF7 cells were passed and grown to 80% confluence in medium.

### 4.3. MCF7 Cells Treatment with B. jararaca Venom

Initially, one milligram of *B. jararaca* venom was dissolved in 1 mL of MCF7 medium. Based on previous results with HUVECs [29], four different concentrations (1, 2, 5, and 10 μg/mL) were tested. We then chose the highest concentration (5 μg/mL) at which no overt phenotypic changes were observed in the MCF7 cells, and added 1mL of this solution to each well on a six-well plate (85.20 mm × 127.80 mm). After that, the cells were incubated for 6 h at 37 °C. A plate containing only cells in 1 mL of media was assayed as control. All experiments were performed in duplicate.

### 4.4. Gene Expression Analysis

The total RNA was extracted from the cells using the RNeasy mini kit (Quiagen, Hilden, Germany, cat. no. 74104) following the manufacturer’s instructions. The sense strand DNA was generated from cRNA, fragmented, and labeled for hybridization to HuGene ST 2.0 array (Affimetrix, lot. 4265888, Ref. 902112, Thermo Fisher Scientific, Waltham, MA, USA). The samples were hybridized to the chips overnight and washed and stained using Affymetrix’s Fluidics Station 450 (P/N 00-0079, Affymetrix, Santa Clara, CA, USA) and the GeneChip Hybridization, Wash and Stain kit (P/N 900720, Affymetrix) following the manufacturer’s instructions. The chips were scanned using Affymetrix’s GeneChip Scanner 3000 7G (p/n 00-0213, Affymetrix, Santa Clara, CA, USA). Four chips were run for the two experimental groups (venom and control) assayed in duplicate, as aforementioned.

### 4.5. Bioinformatics Analysis

The gene expression analysis, to determine changes in transcripts following MCF7 cell treatment with *B. jararaca* in comparison to untreated cells, was carried out as previously described [33]. Furthermore, we also used the C-map software build 02 (https://portals.broadinstitute.org/cmap/) to query the probe sets of significantly differentially expressed genes with the perturbagen signatures present in the C-map database. Initially, we converted the probe sets from HuGene ST 2.0 to HGU133A dataset, which is compatible with the C-map database, using the *Affymetrix* tool that provided a best match between the two chip types.

Afterwards, the algorithm returned a ranked list of all perturbagens found in the C-map database along with scores indicating their relation to the venom. The top-100 positively correlated drugs identified through C-map were submitted to the website Drugbank, available on query (https://www.drugbank.ca/, (accessed on 12 June 2017) [222], to retrieve information about their mechanisms of action. The same was done to the top-20 negatively correlated drugs.

## Figures and Tables

**Table 1:** Summary information on *Bothrops jararaca* venom components.

Protein Class	Associated Activities	Molecular Mass (kDa)	Relative Abundance (%) ^a^
Metalloendopeptidase	Degrades extracelular matrix and coagulation cascade components leading to hemorrhage, edema, inflammation, and necrosis [38,39,40]	20–100	33.6
Serine endopeptidase	Acts on platelet aggregation, blood coagulation, fibrinolysis, complement system, blood pressure, and the nervous system [41,42,43]	20–70	22.8
C-type lectin/C-type lectin-like	Anticoagulant, procoagulant, agonist/antagonist of platelet activation [44]	26–124	18.2
Cysteine-rich secretory protein	Induces inflammatory response and affects the complement system (anaphylatoxins generation) [45,46]	25	8.2
Phospholipase A_2_	Miotoxicity, neurotoxicity, anticoagulant effects [41,47]	12–15	6.3
l-amino acid oxidase	Agonist and antagonist of platelet aggregation; induces apoptosis [48]	110–150	5.0
Snake venom vascular endothelial growth factor	Increases vascular permeability [49,50]	30	1.4
Bradykinin-potentiating- and C-type-natriuretic peptides	Vasodilatation by inhibition of angiotensin-converting enzyme [1,51]	<2.5	1.3
Phosphodiesterase	Pyrimidine and purine release, possibly contributing to the increase of vascular permeability [52,53]	100–130	<1.0
Hyaluronidase	Degrades the hyaluronic acid present in the extracellular matrix, facilitating toxin diffusion [54]	30–80	<1.0
Ecto-5′-nucleotidase	Pyrimidine and purines release, possibly contributing to the increase of vascular permeability [52]	74	<1.0
Metalloendopeptidase inhibitor	Inhibits enzymatic and hemorrhagic activity of snake venom metalloendopeptidases; abundantly found in the snake’s plasma (protective mechanism) [55]	46	<1.0
Disintegrin	Inhibits platelet aggregation [56]	4–15	<1.0
Cobra venom factor ^b^	Activates the complement cascade [57]	149	<1.0
Three-finger toxin ^b^	Neurotoxicity and cardiotoxicity effects by targeting nicotinic and muscarinic acetylcholinesterase receptors, beta-adrenergic receptors, and L-type calcium channels [58,59]	6–8	<1

^a^ [34]; ^b^ Major components in venoms from Elapidae snakes, although identified as minor components in *B. jararaca* venom [34].

**Table 2:** Hypothetical activities that could lead to therapeutical applications (identified by the present work) which have already been reported for snake venoms (or fractions thereof).

Activity	Venom Source	Reference
Antibacterial	*Bothrops jararaca*; *B. asper*; *B. alternatus*; *B. atrox*; *B. pirajai*: *Bothropoides lutzi*	[4,8,9,10,77,78]
Anti-parasatic (trypano-, leishmani-, and plasmodicidal)	*B.jararaca*; *B. moojeni*; *Crotalus adamanteus*; *B. jararacussu*; *B. asper*; *B. pirajai*; *C. durissus collilineatus*; *B. marajoensis*; *B. lutzi*; *C. d. cumanensis*	[4,11,12,13,14,16,77,78,79,80,81,82,83,84,85]
Antihypertensive	*B. jararaca*	[1,2,86]
Antitumor	*B. jararaca*; *Ophiophagus hannah*; *Agkistrodon acutus*; *Bungarus fasciatus*; *B. atrox*; *B. leucurus*; *C. atrox*; *Lachesis muta*; *A. contortrix laticinctus*; *A. halys*; *A. halys pallas*; *B. moojeni*; *B. pirajai*; *Calloselasma rhodostoma*	[87,88,89,90,91]
Antiparkinsonian	*B. atrox*	[92]
Anti-inflammatory and/or analgesic	*Naja naja*; *N. n. atra*; *C. d. terrificus*; *O. hannah*	[20,21,22,23,24,93,94]
Antidiabetic	*C. adamanteus*; *C. vegrandis*; *Bitis nasico*; *C. d. cascavella*; *C. d. terrificus*; *N. kaouthia*; *C. d. collilineatus*	[95,96,97,98,99,100,101]

**Table 3:** C-map hits for antimicrobial drugs, following MCF7 cells incubation with *Bothrops jararaca* venom.

C-Map Name	Dose (nM)	Score ^a^	Up ^b^	Down ^c^	Drug Type
Primaquine	0.9 × 10^4^	0.915	0.429	−0.517	Antiparasite (antimalarian activity)
Tanespimycin	0.1 × 10^4^	0.814	0.161	−0.681	Antineoplastic Antibiotic
Cefalonium	0.9 × 10^4^	0.775	0.250	−0.551	Antibiotic
Chlorhexidine	0.8 × 10^4^	0.743	0.102	−0.667	Antibiotic
Novobiocin	1.0 × 10^5^	0.737	0.056	−0.706	Antibiotic
Clioquinol	1.3 × 10^4^	0.737	0.366	−0.396	Antifungal and antiprotozoal
Erythromycin	0.5 × 10^4^	0.721	0.119	−0.627	Antibiotic
Tetracycline	0.8 × 10^4^	0.710	0.211	−0.523	Antibiotic
Piperacillin	0.7 × 10^4^	0.677	0.130	−0.571	Antibiotic
Ciclacillin	1.2 × 10^4^	0.675	0.069	-0.629	Antibiotic
Halofantrine	0.7 × 10^4^	0.675	0.146	−0.552	Antimalarial
Colistin	0.3 × 10^4^	0.665	0.167	−0.521	Antibiotic
Cefoxitin	0.9 × 10^4^	0.662	0.260	−0.424	Antibiotic
Minocycline	1.1 × 10^4^	0.653	0.143	−0.532	Antibiotic
Azlocillin	0.8 × 10^4^	0.649	0.099	−0.572	Antibiotic
Vancomycin	0.3 × 10^4^	0.646	0.096	−0.572	Antibiotic
Sulfamonomethoxine	1.4 × 10^4^	0.628	0.095	−0.555	Antibiotic
Dicloxacillin	0.8 × 10^4^	0.627	0.097	−0.551	Antibiotic
Hycanthone	1.1 × 10^4^	0.619	0.137	−0.503	Antischistosomal
Ribostamycin	0.7 × 10^4^	0.605	0.126	−0.500	Antibiotic

^a^ Values between +1 and −1 represent the relative strength of a given signature in an instance from the total set of calculated instances; ^b^ values between +1 and −1 represent the absolute enrichment of an up tag-list in a given instance; ^c^ values between +1 and −1 represent the absolute enrichment of a down tag-list in a given instance.

**Table 4:** C-map hits for neuropsychiatric illnesses treatment drugs, following MCF7 cells incubation with *Bothrops jararaca* venom.

C-Map Name	Dose (nM)	Score ^a^	Up ^b^	Down ^c^	Drug Type
Carbamazepine	1.0 × 10^2^	0.803	0.219	−0.611	Anticonvulsant (epilepsy and nerve pain treatment)
Thioridazine	1.0 × 10^4^	0.802	0.241	−0.589	Antipsychotic (schizophrenia treatment)
Prochlorperazine	1.0 × 10^4^	0.787	0.218	−0.596	Antipsychotic (schizophrenia, nonpsychotic anxiety treatment)
Perphenazine	1.0 × 10^4^	0.785	0.260	−0.552	Antipsychotic (schizophrenia treatment)
Metixene	1.2 × 10^4^	0.778	0.302	−0.503	Antiparkinsonian
Pirlindole	1.2 × 10^4^	0.740	0.140	−0.625	Antidepressant
Mianserin	1.3 × 10^4^	0.726	0.226	−0.525	Antidepressant
Lisuride	1.2 × 10^4^	0.722	0.242	−0.505	Antiparkinsonian
Mesoridazine	0.7 × 10^4^	0.721	0.109	−0.637	Antipsychotic (schizophrenia treatment)
Clozapine	1.0 × 10^4^	0.712	0.109	−0.627	Antipsychotic (treatment-resistant schizophrenia)
Trimethadione	2.8 × 10^4^	0.681	0.117	−0.587	Anticonvulsant (seizures treatment)
Zuclopenthixol	0.9 × 10^4^	0.673	0.192	−0.505	Antipsychotic (schizophrenia treatment)
Haloperidol	1.0 × 10^4^	0.669	0.057	−0.635	Antipsychotic (schizophrenia and Huntington’s disease treatment)
Thioproperazine	0.6 × 10^4^	0.658	0.160	−0.520	Antipsychotic (schizophrenia treatment)
Diclofenamide	1.3 × 10^4^	0.631	0.141	−0.511	Anticonvulsant (antiglaucoma, antiepileptic)
Levomepromazine	0.9 × 10^4^	0.627	0.214	−0.434	Antipsychotic (schizophrenia, anxiety treatment)
Fluphenazine	1.0 × 10^4^	0.623	0.284	−0.361	Antipsychotic (psychotic disorders treatment)
Valproic Acid	5.0 × 10^4^	0.622	0.174	−0.469	Anticonvulsant (antiepileptic)
Paroxetine	0.1 × 10^4^	0.604	0.103	−0.522	Antidepressant

^a^ Values between +1 and −1 represent the relative strength of a given signature in an instance from the total set of calculated instances; ^b^ values between +1 and −1 represent the absolute enrichment of an up tag-list in a given instance; ^c^ values between +1 and −1 represent the absolute enrichment of a down tag-list in a given instance.

**Table 5:** C-map hits for cardiovascular disorders treatment drugs, following MCF7 cells incubation with *Bothrops jararaca* venom.

C-Map Name	Dose (nM)	Score ^a^	Up ^b^	Down ^c^	Drug Type
Clopamide	1.2 × 10^4^	0.771	0.126	−0.671	Antihypertensive
Dobutamine	1.2 × 10^4^	0.714	0.126	−0.612	Treatment of heart failure and cardiogenic shock
Amrinone	2.1 × 10^4^	0.709	0.137	−0.596	Vasodilator
Quinidine	1.1 × 10^4^	0.702	0.112	−0.614	Arrhythmias
Sotalol	1.3 × 10^4^	0.679	0.121	−0.582	Arrhythmias
Metolazone	1.1 × 10^4^	0.673	0.115	−0.581	Antihypertensive
Papaverine	1.1 × 10^4^	0.652	0.133	−0.542	Vasodilator
Phenoxybenzamine	1.2 × 10^4^	0.637	0.247	−0.413	Antihypertensive
Midodrine	1.4 × 10^4^	0.625	0.155	−0.492	Antihypotensive
Isoprenaline	1.6 × 10^4^	0.617	0.148	−0.490	Bradycardia
Minoxidil	1.9 × 10^4^	0.608	0.092	−0.537	Vasodilator
Moracizine	0.9 × 10^4^	0.607	0.198	−0.430	Arrhythmias
Hydroflumethiazide	1.2 × 10^4^	0.604	0.131	−0.494	Antihypertensive
Tocainide	1.7 × 10^4^	0.602	0.150	−0.472	Arrhythmias
Practolol	1.5 × 10^4^	0.602	0.152	−0.471	Arrhythmias

^a^ Values between +1 and −1 represent the relative strength of a given signature in an instance from the total set of calculated instances; ^b^ values between +1 and −1 represent the absolute enrichment of an up tag-list in a given instance; ^c^ values between +1 and −1 represent the absolute enrichment of a down tag-list in a given instance.

**Table 6:** C-map hits for anti-inflammatory drugs, following MCF7 cells incubation with *Bothrops jararaca* venom.

C-Map Name	Dose (nM)	Score ^a^	Up ^b^	Down ^c^	Drug type
Sulindac	1.1 × 10^4^	0.854	0.330	−0.553	Anti-inflammatory
Thalidomide	1.0 × 10^5^	0.752	0.145	−0.632	Anti-inflammatory
Oxyphenbutazone	1.2 × 10^4^	0.732	0.258	−0.499	Anti-inflammatory
Tenoxicam	1.2 × 10^4^	0.710	0.107	−0.627	Anti-inflammatory
Epirizole	1.7 × 10^4^	0.700	0.152	−0.572	Anti-inflammatory
Indoprofen	1.4 × 10^4^	0.672	0.071	−0.624	Anti-inflammatory and analgesic
Budesonide	0.9 × 10^4^	0.665	0.126	−0.562	Anti-inflammatory (Crohn’s Disease Treatment)
Methylprednisolone	1.1 × 10^4^	0.663	0.142	−0.544	Anti-inflammatory
Mefenamic Acid	1.7 × 10^4^	0.645	0.104	−0.563	Anti-inflammatory
Felbinac	1.9 × 10^4^	0.642	0.151	−0.513	Anti-inflammatory (analgesic and antipyretic)
Acemetacin	1.0 × 10^4^	0.627	0.116	−0.532	Anti-inflammatory

^a^ Values between +1 and −1 represent the relative strength of a given signature in an instance from the total set of calculated instances; ^b^ values between +1 and −1 represent the absolute enrichment of an up tag-list in a given instance; ^c^ values between +1 and −1 represent the absolute enrichment of a down tag-list in a given instance.

## Supplementary Materials

The following are available online at http://www.mdpi.com/2072-6651/10/2/69/s1, Table S1: Up-regulated MCF7 genes after *Bothrops jararaca* venom treatment, Table S2: Down-regulated MCF7 genes after *Bothrops jararaca* venom treatment, Table S3: Top-100 positively correlated C-map hits for drug-related activities potentially present in *Bothrops jararaca* venom, Table S4: Top-20 negatively correlated C-map hits for drug-related activities potentially present in *Bothrops jararaca* venom, Table S5: Gene expression profiles induced by *Bothrops* venom on MCF7 cells, Table S6: Full signal intensities obtained by *Bothrops jararaca* venom and control (only MCF7 cells), and Table S7: Full C-map hits for drug-related activities identified for *Bothrops jararaca* venom (only MCF7 cells).
